# Structure and Optical Properties of TiO_2_ Films Prepared by Electron Beam Evaporation of Al_2_O_3_-Doped Ti_3_O_5_

**DOI:** 10.3390/ma19081614

**Published:** 2026-04-17

**Authors:** Cheng Peng, Xingqi Wang, Zhixia Shi, Huaying Duan, Bitian Zhang, Yanxi Yin

**Affiliations:** 1 National Engineering Research Center for Environment-Friendly Metallurgy in Producing Premium Non-Ferrous Metals, GRINM Group Corporation Limited, Beijing 101407, China; 2 GRINM Resources and Environment Tech. Co., Ltd., Beijing 101407, China; 3 Beijing Engineering Research Center of Strategic Nonferrous Metals Green Manufacturing Technology, Beijing 101407, China; 4 General Research Institute for Nonferrous Metals, Beijing 100088, China

**Keywords:** Ti_3_O_5_, Al_2_O_3_ doping, electron beam evaporation, TiO_2_ films, optical properties

## Abstract

**Highlights:**

**What are the main findings?**

- Al_2_O_3_ doping stabilizes high-temperature stable λ-Ti_3_O_5_ to room temperature, with complete β→λ phase transition at 5 at% doping content.
- 12.5 at% Al_2_O_3_ doping is determined as the optimal content, balancing stable composition transfer, low extinction coefficient, and moderate refractive index for TiO_2_ films.
- Al_2_O_3_ doping continuously reduces TiO_2_ film refractive index/extinction coefficient, while increasing optical band gap and surface roughness.

**What are the implications of the main findings?**

- The phase transition regulation mechanism via Al^3+^-induced lattice contraction/distortion is revealed, guiding Ti_3_O_5_-based material design.
- This work provides experimental/theoretical support for TiO_2_ film doping modification and high-performance optical filter applications.

**Abstract:**

The crystal structure regulation of Ti_3_O_5_ by Al_2_O_3_ doping and its effect on the optical properties of TiO_2_ films prepared by electron beam evaporation were systematically studied. Ti_3_O_5_ coating materials with different Al_2_O_3_ doping contents (0–50 at%) were prepared by vacuum melting, and the corresponding TiO_2_ films were deposited on K9 glass substrates via electron beam vacuum evaporation. The phase structure, phase transition temperature, chemical composition and optical properties of the materials and films were characterized by XRD, DSC, EDS, XPS, UV-Vis and AFM. Results show that Al_2_O_3_ doping induces the phase transition of Ti_3_O_5_ from a room-temperature stable β-phase to a high-temperature stable λ-phase, with complete transition at 5 at% doping. Al^3+^ with a smaller ionic radius causes lattice contraction and local distortion of Ti_3_O_5_, enabling stabilization at room temperature of the λ-phase. For TiO_2_ films, 12.5 at% doping is the optimal state with the stable composition transfer under this condition. With the increase in Al_2_O_3_ doping content, the refractive index and extinction coefficient of TiO_2_ films decrease continuously, while the optical band gap and surface roughness show an increasing trend. The changes in optical properties are mainly ascribed to the low refractive index of Al_2_O_3_, lattice compressive strain effect and oxygen vacancy passivation induced by Al^3+^. This study clarifies the regulation effect of Al_2_O_3_ doping on Ti_3_O_5_ phase transition and TiO_2_ film optical properties, and provides theoretical basis and experimental reference for the doping modification of TiO_2_ films and their practical applications in consumer electronics and optical filter devices.

## 1. Introduction

TiO_2_ films with excellent optical transparency and chemical stability are widely used as high-refractive-index materials in optical filters, anti-reflection coatings and other optical devices for the visible and near-infrared spectrum. Reactive electron beam evaporation using Ti_3_O_5_ as the coating raw material is an effective method to prepare high-quality TiO_2_ films, which has the advantages of low evaporation temperature, stable oxygen content and easy preparation of stoichiometric and highly reproducible TiO_2_ films [1]. However, Ti_3_O_5_ undergoes a reversible solid-state phase transition from β-phase (room-temperature stable) to λ-phase (high-temperature stable) during heating and cooling. This phase transition causes crystal volume expansion and contraction, leading to material cracking and splashing during evaporation, which seriously affects the surface smoothness and optical performance of the prepared TiO_2_ films.

Doping modification is an effective strategy to stabilize the high-temperature stable λ-Ti_3_O_5_ phase to room temperature and suppress its phase transition. Existing research on Ti_3_O_5_ doping mainly focuses on the preparation and functional application of doped λ-Ti_3_O_5_. Nakamura et al. [2] calculated the binding energy of 54 metal-doped λ-Ti_3_O_5_ and prepared Sc-doped λ-Ti_3_O_5_ for thermal energy storage research. Shen et al. [3] prepared Al-doped λ-Ti_3_O_5_ by carbothermal reduction and studied the effect of Al doping on the electrical resistance of λ-Ti_3_O_5_. Wang et al. [4] investigated the phase evolution law of Mg-doped Ti_3_O_5_ and the formation mechanism of λ-phase. For TiO_2_ film preparation, various methods such as sol–gel, electron beam evaporation and magnetron sputtering have been widely used, and doping modification is often adopted to regulate its optical properties for photocatalysis, photovoltaics and other applications. Ahmad et al. [5] prepared Mg/Ni/Sn-doped TiO_2_ films by sol–gel dip-coating and systematically studied the regulation of metal doping on the structure and optical/electrical properties of TiO_2_ films. Rehman et al. [6] prepared TiO_2_/V_2_O_5_/TiO_2_ multilayer films by electron beam evaporation and analyzed the changes in band gap width and thermal stability. Ogawa et al. [7] studied the effect of oxygen content on the photocatalytic activity of TiO_2_ films prepared by magnetron sputtering.

However, there is a lack of systematic research on the component transfer stability of Al_2_O_3_-stabilized Ti_3_O_5_ coating materials during electron beam vacuum evaporation, as well as the specific influence mechanism of Al ions on the optical band gap, absorption coefficient and surface morphology of TiO_2_ films. In this work, Al_2_O_3_-doped Ti_3_O_5_ coating materials with different doping contents were prepared by vacuum melting, and TiO_2_ films were deposited on K9 substrates by electron beam evaporation. The inhibitory effect of Al_2_O_3_ doping on Ti_3_O_5_ phase transition was clarified, and the influence rule of Al_2_O_3_ doping content on the chemical composition, surface morphology and optical properties of TiO_2_ films was revealed. This study provides a theoretical basis and experimental reference for the optimization of TiO_2_ film preparation process and its application in high-performance optical devices.

## 2. Materials and Methods

### 2.1. Experimental Materials

High-purity TiO_2_, Ti and Al_2_O_3_ powders (4N grade, Sinopharm Group Chemical Reagent Co., Ltd., Shanghai, China) were used as raw materials. The molar ratio of TiO_2_ to Ti powder was fixed at 9:1 to synthesize Ti_3_O_5_. The raw materials were uniformly mixed according to the Al_2_O_3_/TiO_2_ molar ratios of 0, 0.5%, 1.5%, 2.5%, 5%, 12.5% and 50%. Al_2_O_3_-doped Ti_3_O_5_ coating materials were prepared by high-temperature melting in a vacuum furnace at 1800 °C under a vacuum of 10^−1^~10^−2^ Pa.

### 2.2. Preparation of Al_2_O_3_-Doped TiO_2_ Films

Al_2_O_3_-doped TiO_2_ films were prepared by electron beam vacuum evaporation using a ZZS900 type vacuum coating machine (Chengdu Vacuum Machinery Factory, Chengdu, China). K9 glass was used as the substrate (China Southern Glass Co., Ltd., Chengdu, China), and the base vacuum of the coating chamber was pumped to 3 × 10^−3^ Pa. The substrate temperature was set to 100 °C during evaporation, and high-purity oxygen was introduced to control the chamber vacuum at 1 × 10^−2^ Pa. The evaporation parameters were as follows: high voltage 8 KV, evaporation rate 0.3 nm/s (controlled by XTC/3M, INFICON Inc., East Syracuse, NY, USA, and substrate rotation speed 15 r/min to ensure uniform film deposition. The samples were stored in PVC bags and measured within 72 h.

### 2.3. Material Performance Characterization

#### 2.3.1. Material Phase and Phase Transition Temperature Testing

The phase composition of Al_2_O_3_-doped Ti_3_O_5_ was analyzed by an X-ray diffractometer (XRD, D8Advance, Bruker Corporation, Billerica, MA, USA) with Cu Kα monochromatic X-ray source (wavelength 1.54056 Å). The test conditions were: scan range 10–90°, scan rate 5°/min, step size 0.01°. The phase transition temperature of doped Ti_3_O_5_ was characterized by a simultaneous thermal analyzer (DSC, 449F-1, Netzsch-Gerätebau GmbH, Selb, Germany) with thermogravimetry and differential scanning calorimetry functions. The test was carried out in Ar atmosphere with a maximum temperature of 300 °C and a heating rate of 10 °C/min. The chemical composition and element distribution of the materials were analyzed by energy dispersive spectroscopy (EDS, FEI Tecnai G2 F3, Oxford Instruments, Abingdon, UK).

#### 2.3.2. Film Performance Analysis

The transmittance of TiO_2_ films in the ultraviolet to near-infrared region was measured by a UV-visible spectrophotometer (UV2600, Shimadzu Corporation, Kyoto, Japan) with a wavelength range of 300–800 nm. The surface chemical composition and element valence state of the films were analyzed by X-ray photoelectron spectroscopy (XPS, ESCALAB 250Xi, Thermo Fisher Scientific, Waltham, MA, USA). XPS measurements were performed using an Al Kα X-ray source with a photon energy of 1486.6 eV. The photoelectron emission angle was set to 90° relative to the sample surface normal, and the analyzed area was 500 × 500 μm^2^. The samples were placed in the analysis chamber without direct contact with the spectrometer. No sputter-etching was performed prior to analysis. The base pressure in the analysis chamber during measurements was 1 × 10^−9^ mbar, and a charge neutralizer was employed during data acquisition. The survey scan energy range is 0–1350 eV (High-resolution scan: Ti 2p: 448–475 eV, O 1s: 523–541 eV, C 1s: 276–298 eV, Al 2p: 65–85 eV). The surface morphology and roughness of the films were characterized by atomic force microscopy (AFM, Dimension Edge, Bruker Corporation, Billerica, MA, USA).

## 3. Results and Discussion

### 3.1. Regulation of Al_2_O_3_ Doping on Ti_3_O_5_ Crystal Structure and Thermodynamic Phase Transition

**Figure 1:**
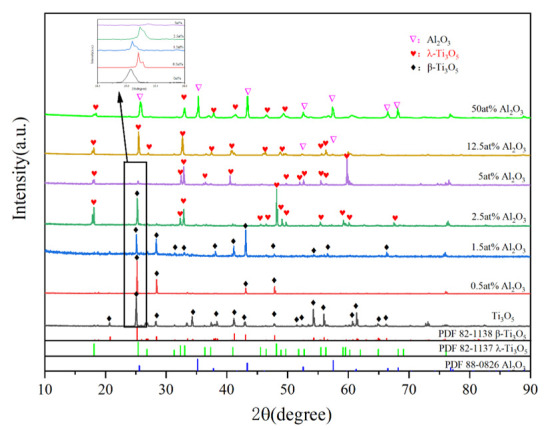
XRD results of Ti_3_O_5_ doped with different Al_2_O_3_ contents.

The XRD results of Ti_3_O_5_ doped with different Al_2_O_3_ contents (Figure 1) show that with the increase in Al_2_O_3_ doping content, the diffraction peak intensity of β-Ti_3_O_5_ gradually decreases, and the λ-Ti_3_O_5_ begins to appear and its intensity gradually increases. The phase composition of Ti_3_O_5_ after different Al_2_O_3_ doping is shown in Table 1.

When the Al_2_O_3_ doping content is 0.5 at% and 1.5 at%, no λ-Ti_3_O_5_ is observed, but the position of the strong diffraction peak of β-Ti_3_O_5_ changes slightly compared with the pure sample, indicating that Al^3+^ has entered the Ti_3_O_5_ lattice and caused lattice distortion. When the doping content reaches 2.5 at%, the λ-Ti_3_O_5_ begins to appear, and most of the λ-phase is stabilized to room temperature. When the doping content is 5 at%, the β-Ti_3_O_5_ completely transforms into the λ-Ti_3_O_5_, realizing the complete room-temperature stabilization of the high-temperature phase. With the further increase in doping content to 12.5 at%, trace Al_2_O_3_ phase precipitates at the grain boundaries, and a large amount of Al_2_O_3_ phase appears when the doping content reaches 50 at%, which is due to the excess Al^3+^ that cannot enter the Ti_3_O_5_ lattice and forms the second phase. The structural parameters of β-Ti_3_O_5_, λ-Ti_3_O_5_, and Al_2_O_3_ are show in Table 2.

Comparing the characteristic peak of β-Ti_3_O_5_ at 2θ = 25.1°, the diffraction peak shifts to a higher diffraction angle with the increase in Al_2_O_3_ doping content. According to Bragg’s law (Formula (1)), the interplanar spacing d decreases with the increase in diffraction angle θ, indicating that Al_2_O_3_ doping causes the lattice contraction of Ti_3_O_5_.

2dsinθ = nλ(1)

where d is the interplanar spacing, θ is the diffraction angle, n is the diffraction order, λ is the X-ray wavelength.

**Figure 2:**
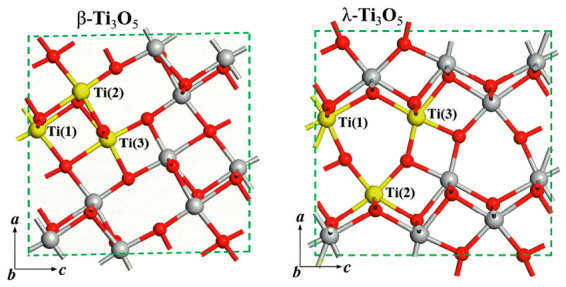
Crystal structure diagrams of β-Ti_3_O_5_ and λ-Ti_3_O_5_.

The crystal structure of Ti_3_O_5_ is constructed with TiO_6_ octahedra as the basic structural unit, which forms a three-dimensional framework through edge and corner sharing (Figure 2) [8]. Ti ions are located at the octahedral center with six-coordinate configuration, and the ionic radii of Ti^3+^ and Ti^4+^ are 0.67 Å and 0.605 Å, respectively, while the ionic radius of doped Al^3+^ is only 0.535 Å, with a radius difference of about 20–25% compared with Ti ions. When Al^3+^ replaces the larger Ti^3+^/Ti^4+^ in the lattice, it causes the contraction of the local TiO_6_ octahedral structure, leading to the micro-adjustment of the lattice parameters of the entire unit cell. The difference in ionic radius leads to the non-uniform change in the bond length and bond angle of the TiO_6_ octahedron (the Al-O bond length is 1.91 Å, shorter than the Ti-O bond length of 2.0–2.1 Å), forming local lattice distortion. This distortion only exists in the short-range surrounding of Al^3+^ and does not damage the integrity of the overall three-dimensional framework under appropriate doping content, thus helping to stabilize the high-temperature stable λ-Ti_3_O_5_ to room temperature.

At the doping levels ranging from 0.5 at% to 5 at%, Al^3+^ is uniformly dispersed at the Ti ion sites in the lattice, and the local lattice distortions cancel each other out, resulting in uniform contraction of the unit cell and basically unchanged crystallinity of the material. At high doping levels, the excess Al^3+^ cannot fully enter the Ti_3_O_5_ lattice, and the undoped Al^3+^ forms the Al_2_O_3_ second phase at the grain boundaries, which is consistent with the XRD results of 12.5 at% and 50 at% doping samples.

**Figure 3:**
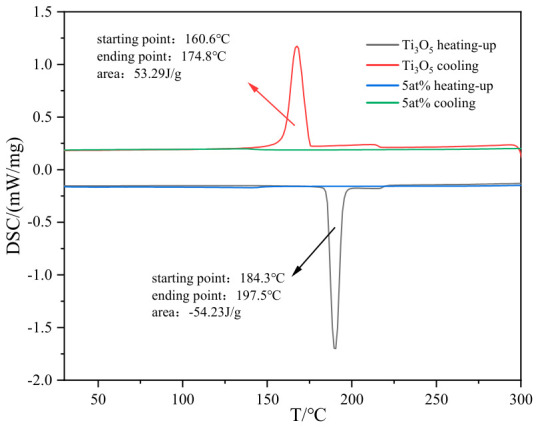
DSC curves of pure Ti_3_O_5_ and 5 at% Al_2_O_3_-doped Ti_3_O_5_.

The DSC analysis results of pure Ti_3_O_5_ and 5 at% Al_2_O_3_-doped Ti_3_O_5_ (Figure 3) show the significant regulation effect of Al_2_O_3_ doping on the phase transition performance of Ti_3_O_5_. Pure Ti_3_O_5_ begins to undergo phase transition at about 184 °C during heating, which is an endothermic process. The β-phase is the low-temperature stable phase, and its transformation to the high-temperature stable λ-phase needs to absorb heat to overcome the phase transition energy barrier and drive the atomic rearrangement (such as the formation of Ti-Ti dimers [9]). The λ-phase is stable at high temperature, and when the temperature decreases, it spontaneously transforms back to the β-phase and releases heat, showing an exothermic peak at 174–160 °C. For the Ti_3_O_5_ material with 5 at% Al_2_O_3_ doping, only a very weak endothermic peak appears at 141.0–151.4 °C, and the signal is close to the baseline, indicating that the phase transition degree of the doped sample is extremely low and the phase composition is stable at room temperature and heating process.

**Figure 4:**
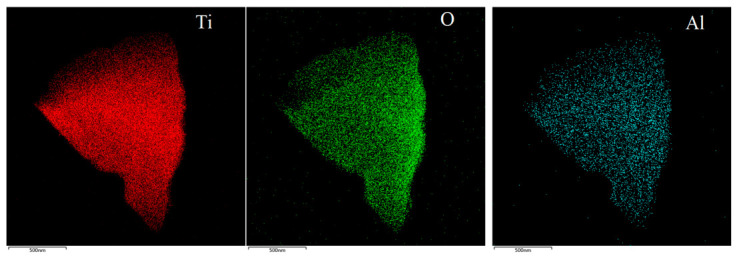

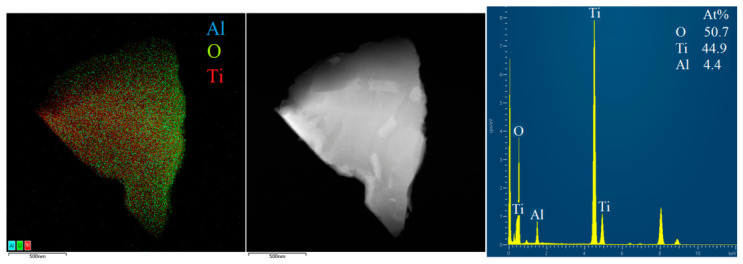
EDS mapping and spectrum of 5 at% Al_2_O_3_-doped Ti_3_O_5_.

The EDS results of 5 at% Al_2_O_3_-doped Ti_3_O_5_ (Figure 4) show that Al element is uniformly distributed in the Ti_3_O_5_ matrix, and the energy spectrum test result of Al/Ti atomic ratio is 1/10, which is close to the theoretical ratio of Al_2_O_3_/TiO_2_ = 5 at%, indicating that Al_2_O_3_ is uniformly doped in the Ti_3_O_5_ material during the vacuum melting process without obvious element segregation.

### 3.2. Film Chemical Composition and Valence State

**Figure 5:**
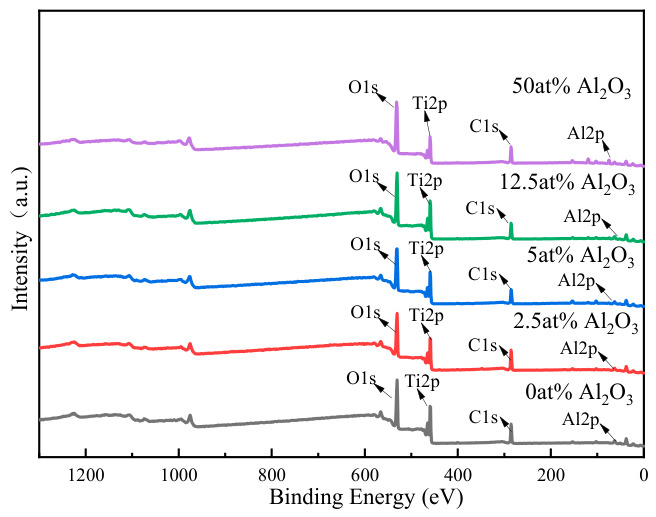
XPS survey spectra of TiO_2_ films with different Al_2_O_3_ doping contents.

To further investigate the effect of Al_2_O_3_ doping on the properties of TiO_2_ thin films, Al_2_O_3_-doped TiO_2_ thin films were prepared. The XPS analysis was used to characterize the surface chemical composition and element valence state of TiO_2_ films prepared from Ti_3_O_5_ with different Al_2_O_3_ doping contents. The XPS full spectrum and characteristic peak curves are shown in Figure 5.

**Figure 6:**
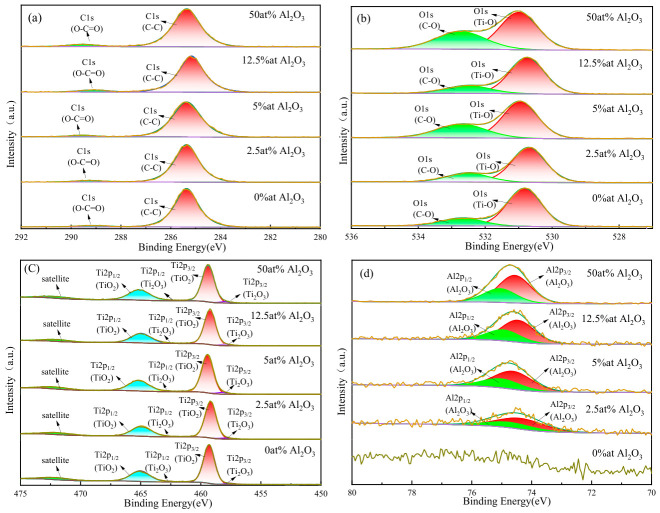
XPS high-resolution spectra of C 1s (**a**), O 1s (**b**), Ti 2p (**c**) and Al 2p (**d**) for TiO_2_ films with different Al_2_O_3_ doping contents.

The binding energy calibration was carefully revised with consideration of the sample work function. The work function of TiO_2_ (ΦSA = 4.22 eV) was taken from the literature [10]. The corrected C 1s reference position was calculated as BE = 289.58 − ΦSA = 285.36 eV. The energy shift was determined from the difference between the measured C 1s binding energy and the corrected BE, and this shift was applied to all core-level spectra. High-resolution spectra of C 1s, O 1s, Ti 2p, and Al 2p were deconvoluted via peak fitting, and the detailed fitting results are presented in Figure 6.

Figure 6a shows the C 1s high-resolution spectra were deconvoluted into two distinct components for all samples. A dominant peak centered at ~285.4 eV, assigned to the C–C bond, which served as the internal reference for charge correction. A weak peak at ~289 eV, attributed to the O–C=O bond from trace surface carbon contamination [11]. No significant shift in the C–C peak position was observed across all doping concentrations, confirming the consistency of charge correction throughout the series.

Figure 6b shows the O 1s spectra were fitted with two components. A main peak at ~530.8 eV, corresponding to lattice oxygen in Ti–O bonds of the TiO_2_ matrix. A secondary peak at ~532.6 eV, assigned to C–O bonds from surface adsorbed oxygen and residual carbon contamination. The Ti–O peak position remained stable with increasing Al_2_O_3_ content, indicating that Al^3+^ doping did not significantly alter the chemical environment of Ti–O bonds in the TiO_2_ lattice.

Figure 6c shows The Ti 2p spectra were deconvoluted into spin–orbit split doublets for Ti^4+^ (TiO_2_) and Ti^3+^ (Ti_2_O_3_). Ti^4+^: Ti 2p_3_/_2_ at ~459 eV and Ti 2p_1_/_2_ at ~464.8 eV, which was the dominant component in all samples, confirming the TiO_2_ phase as the main matrix. Ti^3+^: Ti 2p_3_/_2_ at ~457.7 eV and Ti 2p_1_/_2_ at ~463.5 eV, a minor component attributed to intrinsic oxygen vacancies in TiO_2_. A weak satellite peak was observed at ~472 eV.

Figure 6d shows the Al 2p spectra were only detectable in Al_2_O_3_-doped samples (0 at% Al_2_O_3_ showed no Al signal), and were fitted with spin–orbit split peaks for Al^3+^ in Al_2_O_3_: Al 2p_3_/_2_ at ~74.5 eV and Al 2p_1_/_2_ at ~74.9 eV, confirming that Al was incorporated into the TiO_2_ matrix in the form of Al_2_O_3_. The peak intensity of Al 2p increased monotonically with increasing Al_2_O_3_ content, while the peak position remained stable, verifying the successful and uniform doping of Al_2_O_3_ into the TiO_2_ thin films without the formation of other aluminum-containing impurity phases.

The quantitative XPS results, including atomic percentages of Ti, O, Al, and their respective chemical state ratios, are summarized in Table 3, providing a comprehensive overview of the elemental composition and chemical state evolution with Al_2_O_3_ doping.

The atomic ratios were calculated from XPS peak areas after calibration using the C 1s peak, with normalization to the Al–O–Ti system. As shown in Table 3, at low doping levels (2.5 and 5 at%), the Al_2_O_3_/TiO_2_ ratio in the film deviates from the target, and the Al content is higher than theoretical. This suggests that Al is prone to surface enrichment during electron beam evaporation at low Al_2_O_3_ contents, which can be ascribed to the higher Al–O bond energy, different atomic diffusion rates on the substrate, and preferential adsorption of Al species.

At a raw material doping of 12.5 at%, the film exhibits an Al_2_O_3_/TiO_2_ ratio of 11.6 at%, which is close to the nominal composition, indicating stable component transfer under this condition.

At a high doping level of 50 at%, the measured Al_2_O_3_/TiO_2_ ratio decreases to 40.7 at%, lower than the designed value. This is mainly caused by the preferential evaporation of TiO_2_ from the mixed source. TiO_2_ has higher vapor pressure and lower thermal stability than Al_2_O_3_, leading to more intense evaporation of Ti-containing species. As a result, the relative deposition amount of Ti is increased, thus reducing the Al_2_O_3_ fraction in the film and showing typical non-stoichiometric transfer behavior during multi-component oxide evaporation.

### 3.3. Effect of Al_2_O_3_ Doping on Optical Properties of TiO_2_ Films

#### 3.3.1. Film Transmittance, Refractive Index and Extinction Coefficient

**Figure 7:**
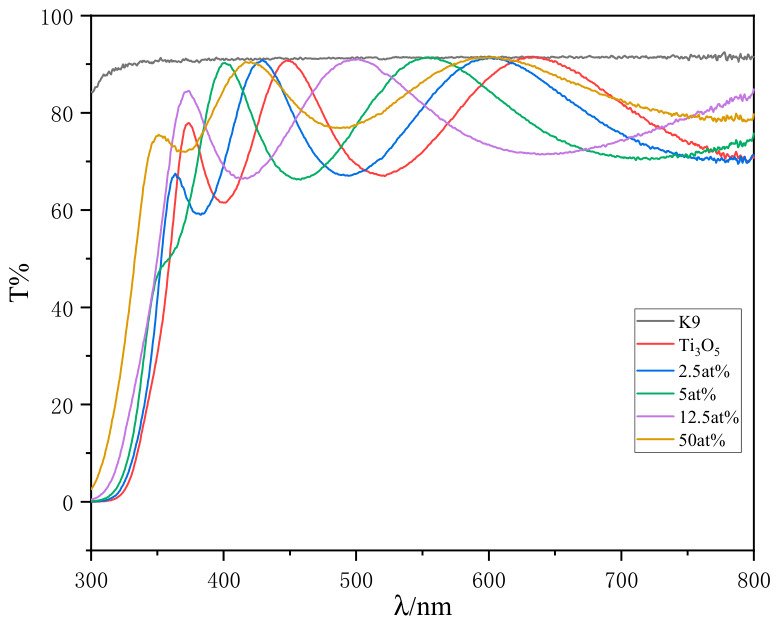
Transmittance curves of Al_2_O_3_-doped TiO_2_ films on K9 glass.

The transmittance curves of TiO_2_ films doped with different Al_2_O_3_ contents in the wavelength range of 300–800 nm (Figure 7) show that all films have high transmittance in the visible region, and the transmittance changes slightly with the increase in Al_2_O_3_ doping content, indicating that Al_2_O_3_ doping does not significantly reduce the light transmittance of TiO_2_ films, which is beneficial to their application in optical devices.

Based on the film transmittance test results, the refractive index (n) and extinction coefficient (k) of the film at 550 nm were fitted and calculated by the Cauchy formula [12] (Formula (2)) and the Exponential model [13] (Formula (3)), and the film thickness was also obtained by fitting. The fitting results are shown in Table 4.

n(λ) = A_0_ + A_1_/λ^2^+ A_2_/λ^4^ (2)

k(λ) = B_1_exp(B_2_λ^−1^) (3)

where n(λ) and k(λ) represent the refractive index and extinction coefficient at the corresponding wavelength λ (nm), respectively; A_0_, A_1_, A_2_, B_1_ and B_2_ are fitting constants.

It can be seen from Table 4 that the refractive index of TiO_2_ films decreases continuously with the increase in Al_2_O_3_ doping content, from 2.277 of the undoped sample to 1.963 of the 50 at% doping sample. For the multi-component mixed dielectric film, the refractive index can be calculated by the formula derived from the Lorentz-Lorenz theory [14] (Formula (4)):

(4) $$n^{2}=\sum_{i=1}^{m}\left(\frac{a_{i}c_{i}n_{i}^{2}}{\rho_{i}}\right)/\sum_{i=1}^{m}\left(\frac{a_{i}c_{i}}{\rho_{i}}\right)$$

where c_i_ is the volume concentration of the i-th medium molecule; a_i_ = 1/(n_i_^2^ + 2); ρ_i_ is the density of the i-th medium; n_i_ is the refractive index of the i-th medium. The refractive index of Al_2_O_3_ is about 1.65, which is much lower than that of TiO_2_ film (2.28). Therefore, with the increase in Al_2_O_3_ content in the film, decreases following the dielectric mixing rule, which is the main reason for the continuous decrease in the refractive index of the doped TiO_2_ film.

For the extinction coefficient of the film, it shows a decreasing trend with the increase in Al_2_O_3_ doping content after a slight increase at low doping content (2.5 at%). Al_2_O_3_ is a wide bandgap insulator with a bandgap of about 7.5 eV, which almost does not absorb photons in the ultraviolet-visible region. When Al_2_O_3_ forms a composite film with TiO_2_, it “dilutes” the light absorption of TiO_2_, leading to the decrease in the extinction coefficient. At the same time, Al_2_O_3_ can fill the oxygen vacancies in the TiO_2_ lattice, passivate the defect states in the film, and reduce the sub-bandgap absorption caused by the defect states, which further reduces the extinction coefficient of the film and improves the optical transmittance.

#### 3.3.2. Film Surface Morphology and Roughness

**Figure 8:**
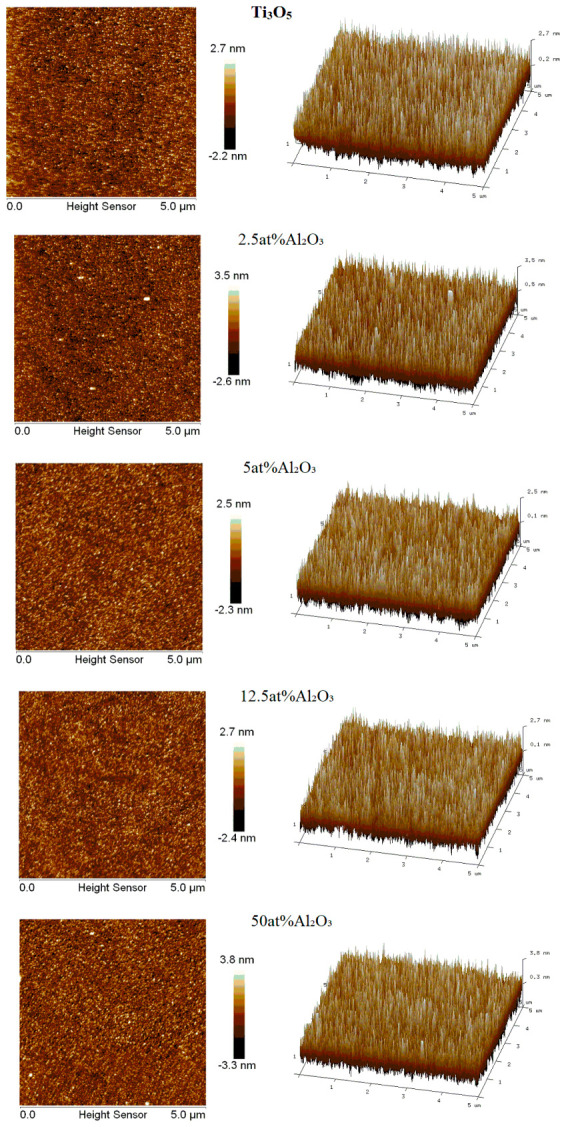
AFM surface morphology images of TiO_2_ films with different Al_2_O_3_ doping contents.

The surface morphology and surface roughness (Rq) of TiO_2_ films with different Al_2_O_3_ doping contents were characterized by AFM (Figure 8), and the test results are shown in Table 5.

The results show that the surface roughness of all TiO_2_ films prepared under this experimental condition is very small (less than 1.1 nm), indicating that the electron beam evaporation method can prepare TiO_2_ films with smooth surface. With the increase in Al_2_O_3_ doping content, the surface roughness of the film gradually increases from 0.608 nm of the undoped sample to 1.01 nm of the 50 at% doping sample. The increase in surface roughness is due to the precipitation of Al_2_O_3_ second phase at the film surface at high doping content, which causes slight unevenness of the film surface. It is worth noting that the extinction coefficient of the film shows a decreasing trend with the increase in doping content, which is opposite to the change trend of surface roughness, indicating that the decrease in the extinction coefficient is not caused by the change in surface roughness, but mainly by the “dilution effect” of Al_2_O_3_ and the passivation of oxygen vacancies.

#### 3.3.3. Film Optical Bandgap

The optical bandgap width of TiO_2_ films was calculated based on the ultraviolet-visible transmittance curve. First, the absorption coefficient α of the film at different wavelengths was calculated by Formula (5) [15]:

α = ln(1/T) × (1/d)(5)

where T is the transmittance of the film at the corresponding wavelength; d is the film thickness.

**Figure 9:**
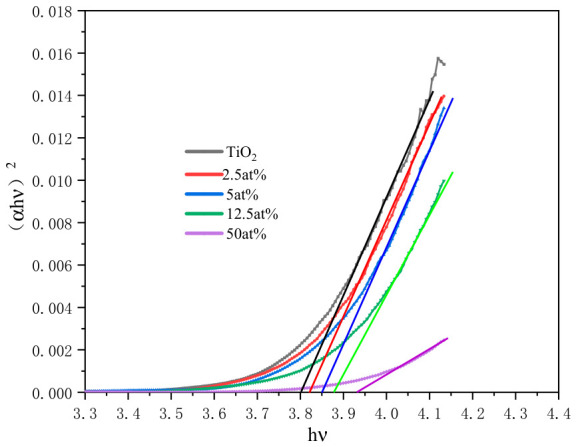
(αhν)^2^ versus hν plots of Al_2_O_3_-doped TiO_2_ films.

For the semiconductor material with direct optical bandgap, the bandgap width Eg of the material was obtained by extrapolating the linear part of the (αhν)^2^-hν curve to the hν axis (where hν is the photon energy, Figure 9). The calculation results of the optical bandgap width of TiO_2_ films with different Al_2_O_3_ doping contents are shown in Table 6.

It can be seen from Table 5 that the optical bandgap width of TiO_2_ films shows an obvious increasing trend with the increase in Al_2_O_3_ doping content, from 3.80 eV of the undoped sample to 3.93 eV of the 50 at% doping sample, showing a blue shift in the bandgap. The bandgap width of the pure TiO_2_ film prepared by electron beam evaporation is 3.80 eV, which is larger than the bulk bandgap width of rutile TiO_2_ (3.4 eV), which is mainly due to the quantum size effect of the nanometer-sized TiO_2_ film [16]. The quantum size effect of the semiconductor film can be described by Formula (6) [17]:

(6) $$E_{g}(\mathrm{QD})=E_{g}(\mathrm{bulk})+\left(\frac{h^{2}}{8R^{2}}\right)\left(\frac{1}{m_{c}}+\frac{1}{m_{h}}\right)-\frac{1.8e^{2}}{4\pi \varepsilon_{0}\varepsilon R}$$

where *Eg* (QD) is the bandgap of quantum dots; *Eg*(bulk) is the bandgap of bulk semiconductor; *h* is Planck’s constant; *R* is the radius of quantum dots; mc and mh are the effective mass of electrons and holes, respectively; *e* is the elementary charge; *ε_0_* is the vacuum permittivity; *ε* is the relative permittivity of the semiconductor material. When the particle size of the semiconductor is small, the quantum confinement effect is dominant, and the positive contribution of the quantum confinement energy term is much greater than the negative contribution of the Coulomb attraction energy term, leading to the increase in the bandgap width of the nanometer-sized film compared with the bulk material.

The further increase in the bandgap width of the TiO_2_ film caused by Al_2_O_3_ doping is mainly due to the lattice compression and strain effect [16]. The ionic radius of Al^3+^ (0.535 Å) is smaller than that of Ti^4+^ (0.605 Å) in the TiO_2_ lattice. When Al^3+^ replaces Ti^4+^ in the TiO_2_ lattice, it causes the contraction of the TiO_6_ octahedral framework and produces compressive strain in the lattice. This compressive strain changes the band structure of TiO_2_, usually making the minimum of the conduction band move upward and/or the maximum of the valence band move downward, thus leading to the broadening of the optical bandgap width of the film, which is the main reason for the blue shift in the bandgap of the doped TiO_2_ film.

## 4. Conclusions

Al_2_O_3_ doping effectively stabilizes λ-Ti_3_O_5_ at room temperature with complete β→λ phase transition at 5 at%. The composition transfer is most stable at 12.5 at% doping. With increasing Al_2_O_3_ content, the refractive index and extinction coefficient decrease, while the band gap and surface roughness increase. These changes originate from the low refractive index of Al_2_O_3_, lattice compressive strain, and oxygen vacancy passivation. This work provides a reliable strategy for preparing stable, high-quality TiO_2_ optical thin films.

## Figures and Tables

**Table 1:** Phase composition of Ti_3_O_5_ after doping with different contents of Al_2_O_3_.

Al_2_O_3_ Doping Content/at%	Phase Composition
0	β-Ti_3_O_5_
0.5	β-Ti_3_O_5_
1.5	β-Ti_3_O_5_
2.5	β-Ti_3_O_5_ + λ-Ti_3_O_5_
5	λ-Ti_3_O_5_
12.5	λ-Ti_3_O_5_ + trace Al_2_O_3_
50	λ-Ti_3_O_5_ + Al_2_O_3_

**Table 2:** Structural parameters of β-Ti_3_O_5_, λ-Ti_3_O_5_, and Al_2_O_3_ phases.

Phase	Crystal Structure	Space Group	a	b	c	β	V
β-Ti_3_O_5_	Monoclinic	C2/m	9.7568	3.8008	9.4389	91.547	349.9
λ-Ti_3_O_5_	Monoclinic	C2/m	9.8261	3.7894	9.9694	91.258	371.12
Al_2_O_3_	Hexagonal	R-3c	4.7607	4.7607	12.997	120	255.1

The crystal structure data according to PDF cards, β-Ti_3_O_5_: No. 82-1138, λ-Ti_3_O_5_: No. 82-1137, Al_2_O_3_: No. 88-0826.

**Table 3:** Chemical composition of TiO_2_ films prepared from Ti_3_O_5_ doped with different contents of Al_2_O_3_.

Sample	Peak	Position/eV	FWHM/eV	Assignment	Atomic Ratio/at%	Al_2_O_3_/TiO_2_ at% (Calculate)
0 at% Al_2_O_3_	C 1s	285.36	1.1	Adventitious C	/	0
	O1s	530.83	1.21	Lattice O (Ti–O/Al–O)	46.27	
	O1s-2	532.7	1.44	O-C=O	11.57	
	Ti2p_3/2_-1	459.31	1.15	Ti^4+^	20.48	
	Ti2p_1/2_-1	465.02	1.94	Ti^4+^	18.88	
	Ti2p_3/2_-2	457.58	1.09	Ti^3+^	0.53	
	Ti2p_1/2_-2	462.84	1.42	Ti^3+^	0.38	
	Ti2p	472.03	2.81	satellite, Ti^4+^	1.9	
2.5 at% Al_2_O_3_	C 1s	285.36	1.14	Adventitious C	/	6.1
	Al2p_3/2_	74.35	1.85	Al^3+^ (Al_2_O_3_)	2.45	
	Al2p_1/2_	74.74	1.85	Al^3+^ (Al_2_O_3_)	2.45	
	O1s	530.7	1.19	Lattice O (Ti–O/Al–O)	43.78	
	O1s-2	532.52	1.44	O-C=O	13.62	
	Ti2p_3/2_-1	459.2	1.11	Ti^4+^	18.02	
	Ti2p_1/2_-1	464.92	1.86	Ti^4+^	16.61	
	Ti2p_3/2_-2	457.89	1.29	Ti^3+^	0.72	
	Ti2p_1/2_-2	462.99	2.16	Ti^3+^	0.66	
	Ti2p	471.88	2.78	satellite, Ti^4+^	1.69	
5 at% Al_2_O_3_	C 1s	285.36	1.34	Adventitious C	/	10.7
	Al2p_3/2_	74.62	1.5	Al^3+^ (Al_2_O_3_)	3.85	
	Al2p_1/2_	75.01	1.5	Al^3+^ (Al_2_O_3_)	3.85	
	O1s	530.95	1.24	Lattice O (Ti–O/Al–O)	39.45	
	O1s-2	532.71	1.73	O-C=O	20.69	
	Ti2p_3/2_-1	459.43	1.11	Ti^4+^	15.29	
	Ti2p_1/2_-1	465.16	1.87	Ti^4+^	14.09	
	Ti2p_3/2_-2	472.05	1.12	Ti^3+^	0.73	
	Ti2p_1/2_-2	458.16	1.88	Ti^3+^	0.68	
	Ti2p	463.47	2.65	satellite, Ti^4+^	1.37	
12.5 at% Al_2_O_3_	C 1s	285.36	1.18	Adventitious C	/	11.6
	Al2p_3/2_	74.46	1.29	Al^3+^ (Al_2_O_3_)	4.68	
	Al2p_1/2_	74.95	1.29	Al^3+^ (Al_2_O_3_)	4.68	
	O1s	530.75	1.23	Lattice O (Ti–O/Al–O)	43.06	
	O1s-2	532.52	1.54	O-C=O	11.83	
	Ti2p_3/2_-1	459.24	1.12	Ti^4+^	17.07	
	Ti2p_1/2_-1	464.95	1.88	Ti^4+^	15.73	
	Ti2p_3/2_-2	457.96	1.1	Ti^3+^	0.74	
	Ti2p_1/2_-2	463.07	1.85	Ti^3+^	0.68	
	Ti2p	471.94	2.72	satellite, Ti^4+^	1.53	
50 at% Al_2_O_3_	C 1s	285.36	1.27	Adventitious C	/	40.7
	Al2p_3/2_	74.57	1.27	Al^3+^ (Al_2_O_3_)	13.72	
	Al2p_1/2_	75.06	1.27	Al^3+^ (Al_2_O_3_)	13.72	
	O1s	530.99	1.35	Lattice O (Ti–O/Al–O)	32.56	
	O1s-2	532.73	1.75	O-C=O	20.04	
	Ti2p_3/2_-1	459.39	1.2	Ti^4+^	9.69	
	Ti2p_1/2_-1	465.1	2.01	Ti^4+^	8.93	
	Ti2p_3/2_-2	457.96	1.05	Ti^3+^	0.33	
	Ti2p_1/2_-2	463.06	1.76	Ti^3+^	0.3	
	Ti2p	471.96	2.84	satellite, Ti^4+^	0.72	

**Table 4:** Optical constants of TiO_2_ films at 550 nm with different contents of Al_2_O_3_.

Evaporation Material	*n*	k	Film Thickness/nm	Root Mean Square Error
Ti_3_O_5_	2.277	7.34 × 10^−4^	283	0.161
2.5at% Al_2_O_3_-doped Ti_3_O_5_	2.262	8.55 × 10^−4^	268	0.150
5at% Al_2_O_3_-doped Ti_3_O_5_	2.246	6.89 × 10^−4^	247	0.115
12.5at% Al_2_O_3_-doped Ti_3_O_5_	2.180	6.06 × 10^−4^	225	0.107
50at% Al_2_O_3_-doped Ti_3_O_5_	1.963	2.72 × 10^−4^	306	0.162

**Table 5:** Surface Roughness of TiO_2_ films with different Al_2_O_3_ doping contents.

Material	Roughness Rq/nm	Ra/nm	Rmax/nm
Ti_3_O_5_	0.608	0.480	7.49
2.5at% Al_2_O_3_-doped Ti_3_O_5_	0.648	0.496	8.68
5at% Al_2_O_3_-doped Ti_3_O_5_	0.695	0.548	8.83
12.5at% Al_2_O_3_-doped Ti_3_O_5_	0.709	0.558	8.26
50at% Al_2_O_3_-doped Ti_3_O_5_	1.01	0.800	10.4

**Table 6:** Optical band gap of TiO_2_ thin films with different Al_2_O_3_ doping contents.

Material	Film Thickness d/nm	Optical Band Gap Eg/eV
Ti_3_O_5_	283	3.80
2.5 at% Al_2_O_3_-doped Ti_3_O_5_	268	3.82
5 at% Al_2_O_3_-doped Ti_3_O_5_	247	3.85
12.5 at% Al_2_O_3_-doped Ti_3_O_5_	225	3.87
50 at% Al_2_O_3_-doped Ti_3_O_5_	306	3.93

## Data Availability

The original contributions presented in this study are included in the article. Further inquiries can be directed to the corresponding authors.

## Abbreviations

The following abbreviations are used in this manuscript:

XRD	X-ray diffractometer
DSC	Differential Scanning Calorimetry
EDS	Energy Dispersive Spectroscopy
XPS	X-ray Photoelectron Spectroscopy
AFM	Atomic Force Microscopy
UV-Vis	Ultraviolet-visible spectrophotometry
